# 3D CNTs/Graphene‐S‐Al_3_Ni_2_ Cathodes for High‐Sulfur‐Loading and Long‐Life Lithium–Sulfur Batteries

**DOI:** 10.1002/advs.201800026

**Published:** 2018-05-10

**Authors:** Zeqing Guo, Huagui Nie, Zhi Yang, Wuxing Hua, Chunping Ruan, Dan Chan, Mengzhan Ge, Xi'an Chen, Shaoming Huang

**Affiliations:** ^1^ Nanomaterials & Chemistry Key Laboratory Wenzhou University Wenzhou 325027 China; ^2^ School of Material and Energy Guangdong University of Technology Guangzhou 510006 China

**Keywords:** Al_3_Ni_2_, graphene, high areal sulfur loading, lithium–sulfur batteries, polysulfides

## Abstract

Lithium–sulfur batteries suffer from poor cycling stability at high areal sulfur loadings (ASLs) mainly because of the infamous shuttle problem and the increasing diffusion distance for ions to diffuse along the vertical direction of the cathode plane. Here, a carbon nanotube (CNT)/graphene (Gra)‐S‐Al_3_Ni_2_ cathode with 3D network structure is designed and prepared. The 3D network configuration and the Al in the Al_3_Ni_2_ provide an efficient channel for fast electron and ion transfer in the three dimensions, especially along the vertical direction of the cathode. The introduction of Ni in the Al_3_Ni_2_ is able to suppress the shuttle effect via accelerating reaction kinetics of lithium polysulfide species conversion reactions. The CNT/Gra‐S‐Al_3_Ni_2_ cathode exhibits ultrahigh cycle‐ability at 1 C over 800 cycles, with a capacity degradation rate of 0.055% per cycle. Additionally, having high ASLs of 3.3 mg cm^−2^, the electrode delivers a high reversible areal capacity of 2.05 mA h cm^−2^ (622 mA h g^−1^) over 200 cycles at a higher current density of 2.76 mA cm^−2^ with high capacity retention of 85.9%. The outstanding discharge performance indicates that the design offers a promising avenue to develop long‐life cycle and high‐sulfur‐loading Li–S batteries.

## 1 Introduction

Lithium–sulfur (Li–S) batteries are one of the most promising energy storage devices, owing to their superior theoretical specific capacity (1675 mA h g^−1^) and high specific energy (2600 Wh kg^−1^) compared with other state‐of‐the‐art lithium–ion batteries. This enhanced performance is based on the complete conversion of sulfur via reaction with lithium metal to form lithium sulfide (Li_2_S).^1^, ^2^ The sulfur used as the cathode material in these batteries has many advantages, including natural abundance, low cost, minimal environmental impact, high biocompatibility, and low toxicity.^3^, ^4^ However, the commercial application of Li–S batteries is still impeded by several critical issues.^5^ The primary challenges are the insulating nature of sulfur, significant volume increases of the cathode, and the dissolution of intermediate lithium polysulfide species (LiPSs) into the electrolyte. The latter not only results in low utilization of the sulfur but also provides a “shuttle of polysulfide” between the electrodes, leading to reduced coulombic efficiency, high self‐discharge, and rapid fading of capacity.^6^, ^7^

To address these issues, many conductive host substances have been employed to confine sulfur and to accommodate intermediate LiPSs. These include various carbon‐based materials (e.g., graphene,^8^, ^9^ carbon nanotubes,^10^ and porous carbon^11^, ^12^), conductive polymers (e.g., polypyrrole,^13^ polyacrylonitrile,^14^ and polyaniline^15^), transition metal oxides,^16^, ^17^, ^18^ transition metal dichalcogenides,^19^, ^20^ and metal‐organic framework based materials.^21^, ^22^ Although the encapsulation strategy has been confirmed to be an effective means of controlling the dissolution of LiPSs, the improved performance that results from this approach is still far from satisfactory, especially at high areal sulfur loadings (ASLs).^23^ This poor performance can be attributed to the constant generation and accumulation of LiPSs in the electrolyte at high ASLs. This process makes it more difficult for ions to diffuse through the electrolyte due to the increasing diffusion distance along the vertical direction of the cathode plane. Recently, various substances, including CoS_2_,^24^ Au,^25^ Ni,^26^ and Pt,^27^ have been employed to promote the efficient transformation of LiPSs and so suppress the shuttle effect in Li–S batteries.^28^ Our own group has also developed a strategy in which dithiothreitol acts as a polysulfide scission reagent in the Li–S system, and this approach has been shown to significantly improve both capacity retention and long‐term cycle stability.^29^ However, despite these significant successes, the performance improvement obtained by simply introducing functional molecules is still limited. As such, the design and development of novel structures that not only promote the efficient transformation of LiPSs but also increase the rate of transmission of ions would be beneficial. Aluminum (Al) foils are frequently used as current collectors for cathodes in lithium–ion and Li–S batteries because of the excellent electronic transmission performance of Al and the mature manufacturing base.^30^ However, Al foil enhances the delivery of electrons only over a 2D plane. It would therefore be of interest to determine whether or not employing aluminum or its alloys in a Li–S system can promote electron transport along the vertical direction of the cathode.

Based on the above, we designed a Li–S battery cathode having a 3D network configuration composed of carbon nanotubes (CNTs), graphene (Gra), and S coupled with Al_3_Ni_2_ (that is, CNTs/Gra‐S‐Al_3_Ni_2_). It was anticipated that the 3D CNTs/Gra network and the introduction of Al in the form of Al_3_Ni_2_ would provide efficient channels for rapid electron and ion transfer, especially along the vertical direction of the cathode. The Ni in the Al_3_Ni_2_ is also able to rapidly eliminate accumulated LiPSs by increasing the rates of the LiPSs conversion reactions. As a result of the aforementioned merits, the optimized electrode generated a much higher initial discharge capacity of 1401 mA h g^−1^ at a current of 0.2 C. This unit also exhibited an increased reversible capacity of 496 mA h g^−1^ when cycled at 1 C over 800 cycles, with only a 0.055% average capacity decay per cycle. Increasing the ASLs to 3.30 mg cm^−2^ resulted in the electrode maintaining a high discharge capacity of 622 mA h g^−1^ after 200 cycles at a current density of 2.76 mA cm^−2^, in conjunction with a high capacity retention of 85.9%.

## 2 Results and Discussion

**Figure 1:**
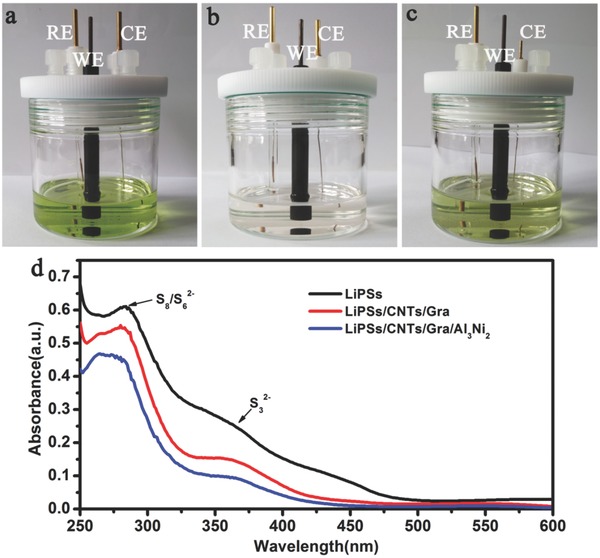
a) Digital photograph of three‐electrode CV test device. Digital photograph of three‐electrode CV test for cycled b) CNTs/Gra/Al_3_Ni_2_ and c) CNTs/Gra. d) UV–vis absorption spectra of the LiPS solution and the solution of CNTs/Gra, CNTs/Gra/Al_3_Ni_2_ electrodes after CV tests.

In this study, commercial Al_3_Ni_2_ (see Figures S1–S3, Supporting Information) was selected as the functional material to improve the Li–S battery performance. The electrocatalytic properties of Al_3_Ni_2_ in the presence of LiPSs were first assessed by cyclic voltammetry (CV) with either CNTs/Gra‐ or CNTs/Gra/Al_3_Ni_2_‐modified glassy carbon (GC) working electrodes in a three‐electrode assembly (**Figure** **1**a) in conjunction with a LiPSs solution.^29^ The CV data were acquired over the voltage range from −2.0 to 1.0 V (Figure S4a–c, Supporting Information). While performing a cycling test with the CNTs/Gra/Al_3_Ni_2_ electrode, the color of the solvent phase was observed to rapidly transition from golden to colorless (Figure 1b), while the solution used with the CNTs/Gra electrode was only slightly decolored (Figure 1c). UV–vis spectroscopy was also employed to further assess the interactions between the Al_3_Ni_2_ and LiPSs. As shown in Figure 1d, a sharp peak is discernible at 280 nm, attributed to S_8_^2−^ and S_6_^2−^, in all three cases (that is, the LiPSs solution on its own and the solution following CV with either electrode). However, the solution obtained in conjunction with the CNTs/Gra/Al_3_Ni_2_ cathode generated the lowest absorbance at 280 nm, and showed almost no peak at 358 nm (due to S_3_^2−^ species).^29^, ^31^ Figure S4a (Supporting Information) provides the voltammograms obtained from the second and tenth cycles for the two electrodes. Both electrodes generated almost have the same redox current density after two cycles. After ten cycles, the current density of the CNTs/Gra electrode was almost unchanged, while that of the CNTs/Gra/Al_3_Ni_2_ was significantly decreased. The aforementioned visual observations and UV–vis spectra suggest that the Al_3_Ni_2_ rapidly eliminated accumulated LiPSs by accelerating the redox reactions of these species.

**Figure 2:**
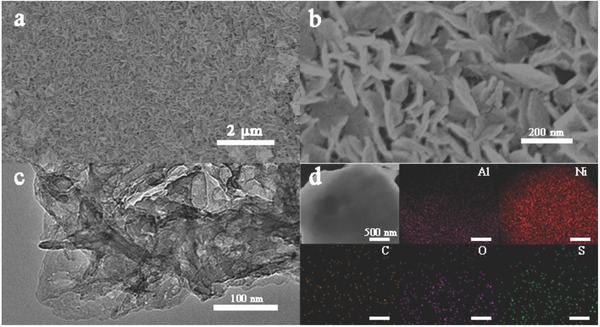
a,b) Low‐ and high‐magnification SEM images of CNTs/Gra/Al_3_Ni_2_ electrode after ten cycles of CV test (CNTs/Gra/Al_3_Ni_2_‐10cyc). c) TEM images of CNTs/Gra/Al_3_Ni_2_‐10cyc. d) STEM and corresponding elemental mapping of CNTs/Gra/Al_3_Ni_2_‐10cyc.

**Figure** **2**a provides a low‐magnification scanning electron microscopy (SEM) image of the CNTs/Gra/Al_3_Ni_2_ specimen after the tenth CV cycle (CNTs/Gra/Al_3_Ni_2_‐10cyc) over a wide area, and shows the presence of uniformly distributed nanosheets. The high‐magnification SEM image (Figure 2b) more clearly demonstrates that this material consisted of numerous interconnected flower‐like nanosheets. These sheets would be expected to have highly active surfaces and thus could promote rapid electron and ion transfer, especially along the vertical direction of the electrode.^32^ The microstructure of this sample was further analyzed by transmission electron microscopy (TEM), as shown in Figure 2c. The highlighted dots in the scanning transmission electron microscopy (STEM) image and the corresponding elemental maps in Figure 2d confirm the uniform spatial distribution of nanoparticles containing Al, Ni, C, O, and S atoms in the CNTs/Gra/Al_3_Ni_2_ after 10 cycles, suggesting that the Al_3_Ni_2_ would be expected to effectively interact with the LiPSs.

**Figure 3:**
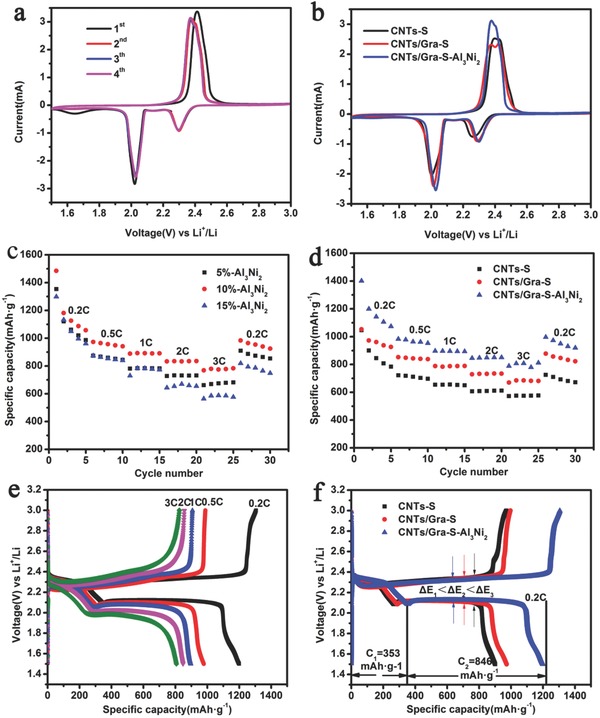
The electrochemical performance of Li–S batteries. a) The first four cycles of CV curves for CNTs/Gra‐S‐Al_3_Ni_2_ cathode. b) The second cycle of CV curves for CNTs‐S, CNTs/Gra‐S, and CNTs/Gra‐S‐Al_3_Ni_2_ cathodes. c) The rate performance of CNTs/Gra‐S‐Al_3_Ni_2_ cathodes with different mass ratio of Al_3_Ni_2_. d) The rate performance of CNTs‐S, CNTs/Gra‐S, and CNTs/Gra‐S‐Al_3_Ni_2_ cathodes, the areal loading amount of sulfur in the rate test is 0.8 mg cm^−2^. e) Galvanostatic charge−discharge profiles of the CNTs/Gra‐S‐Al_3_Ni_2_ cathode at various rates. f) Galvanostatic charge−discharge profiles of the CNTs‐S, CNTs/Gra‐S, and CNTs/Gra‐S‐Al_3_Ni_2_ cathodes at 0.2 C.

The electrochemical performances of CNTs‐S, CNTs/Gra‐S, and CNTs/Gra‐S‐Al_3_Ni_2_ cathodes were compared using 2025 coin cells. The details regarding the fabrication of the CNTs/Gra‐S‐Al_3_Ni_2_ cathode are provided in the Experimental Section. **Figure** **3**a presents typical CV curves generated by the CNTs/Gra‐S‐Al_3_Ni_2_ cathode during the first four cycles within a potential window of 1.5–3 V at a scan rate of 0.1 mV s^−1^. In the first cathodic scan, two well‐defined reduction peaks appear at 2.29 and 2.02 V, corresponding to the transition from elemental S to soluble long‐chain LiPSs and the further reduction of the higher LiPSs species (4 < *n* < 8) to lower species (Li_2_S*_n_*, *n* ≤ 2), respectively. The anodic scan produced a large oxidation peak at 2.41 V, attributed to the conversion of Li_2_S_2_ and Li_2_S to elemental S.^2^, ^20^, ^29^, ^33^, ^34^, ^35^ The negative shift in the oxidation peaks between the first and second cycles is ascribed to the rearrangement of active S from its original positions to more energetically stable sites.^29^, ^34^ Compared with the CV curves of the CNTs‐S and CNTs/Gra‐S cathodes (Figure S7, Supporting Information), the almost overlapping CV curves for the CNTs/Gra‐S‐Al_3_Ni_2_ cathode in the subsequent three cycles suggest that the latter possessed excellent reversible electrochemical stability.

Figure 3b shows the second cycle CV curves of the three cathodes. It is worth emphasizing that the collection coefficient (the ratio of the peak area associated with the formation of Li_2_S, at ≈2.0 V, to that for the formation of LiPSs, at ≈2.4 V) for the dissolved LiPSs at the cathode had values of 2.63, 2.66, and 2.75 for the CNTs‐S, CNTs/Gra‐S, and CNT/Gra‐S‐Al_3_Ni_2_ specimens, respectively. This result indicates that the Al_3_Ni_2_ promoted the rapid conversion of LiPSs and effectively inhibited the shuttle effect.^34^ The data in Table S1 (Supporting Information) also demonstrate that, compared with the CNTs‐S and CNTs/Gra‐S units, the CNTs/Gra‐S‐Al_3_Ni_2_ cathode exhibited the lowest degree of voltage hysteresis (Δ*V*), which suggests a highly facile electrochemical redox reaction and low resistance.^29^

Figure 3c summarizes the rate performances of CNTs/Gra‐S‐Al_3_Ni_2_ cathodes with different Al_3_Ni_2_ contents. An Al_3_Ni_2_ level of 10 wt% evidently produced the highest discharge capacity at various rates. Typical charge–discharge profiles (Figure S6, Supporting Information) also support the observations in Figure 3c. For this reason, the CNTs/Gra‐S‐Al_3_Ni_2_ cathode materials used in subsequent work all contained 10 wt% Al_3_Ni_2_ unless otherwise noted. The rate performance of each of the three cathodes was investigated at various rates, as shown in Figure 3d. Compared with the other two batteries, the CNTs/Gra‐S‐Al_3_Ni_2_ battery delivered a much greater discharge capacity at each rate. Furthermore, when the rate was increased from 0.2 C to 0.5, 1, 2, and 3 C (1 C = 1675 mA g^−1^), the battery exhibited discharge capacities of 1401, 953, 894, 850 and 812 mA h g^−1^, respectively. Interestingly, abruptly switching the rate back to 0.2 C, the CNTs/Gra‐S‐Al_3_Ni_2_ recovered the majority of the original discharge capacity. It is therefore evident that the CNTs/Gra‐S‐Al_3_Ni_2_ battery possessed excellent reversible capacity at various rates. Figure 3e presents the results of galvanostatic charge/discharge trials with the CNTs/Gra‐S‐Al_3_Ni_2_ cathode at different rates from 0.2 to 3 C. The discharge profiles display two typical plateaus of the Li–S system, which are consistent with the CV curves. In addition, these charge/discharge plateaus are retained even at increased rates, suggesting high electrical conductivity and improved charge transfer kinetics through the cathode.^8^, ^36^ Figure 3f shows the charge/discharge profiles acquired at a constant rate of 0.2 C for the three cathodes. The CNTs/Gra‐S‐Al_3_Ni_2_ cathode exhibits the lowest degree of electrochemical polarization (that is, the lowest voltage hysteresis, Δ*E*), which is consistent with the polarized voltage (ΔV) seen in the CV data (Table S1, Supporting Information). Based on these data, it appears that the electrons and ion transport kinetics are improved in the CNTs/Gra‐S‐Al_3_Ni_2_ cathode.^30^

**Figure 4:**
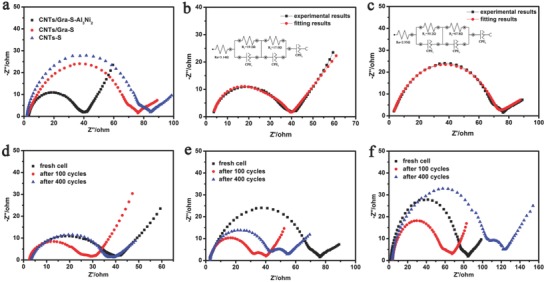
a) Electrochemical impedance spectroscopy of the three cathodes. The Nyquist EIS and corresponding equivalent circuits for b) CNTs/Gra‐S‐Al_3_Ni_2_ and c) CNTs/Gra‐S cathodes. EIS plots of d) CNTs/Gra‐S‐Al_3_Ni_2_, e) CNTs/Gra‐S, and f) CNTs‐S cathodes before and after cycling at rate of 1 C.

Electrochemical impedance spectroscopy (EIS) was also employed to confirm the above results and **Figure** **4**a presents the Nyquist plots of Li–S batteries with three fresh cathodes. The plot obtained with the CNTs/Gra‐S‐Al_3_Ni_2_ cathode exhibits a typical semicircle in the high‐medium frequency region, corresponding to the charge‐transfer process, and an inclined line at the low frequency region, corresponding to Warburg impedance, which is related to the diffusion of lithium ions into the electrode.^37^, ^38^, ^39^ These plots were fitted based on two equivalent circuit diagrams (Figure 4b,c). According to the fitting results, the CNTs/Gra‐S‐Al_3_Ni_2_ cathode had a lower charge‐transfer resistance (40.1 Ω) than the CNTs/Gra‐S cathode (76.3 Ω). The EIS data in Figure 4d–f confirm that the CNTs/Gra‐S‐Al_3_Ni_2_ cathode (Figure 4d) showed the lowest charge‐transfer resistance (*R*_ct_) among the three cathodes, both fresh and after 100 or 400 cycles at 1 C. According to these EIS results, the CNTs/Gra‐S‐Al_3_Ni_2_ cathode had the lowest *R*_ct_ value, demonstrating higher utilization of the active material and indicating enhanced charge and ion transfer, possibly due to their high conductivity.^40^, ^41^

**Figure 5:**
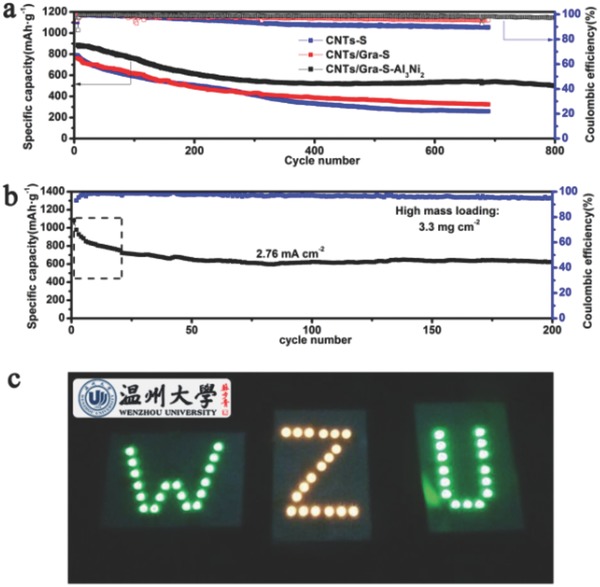
a) Long cycling stability of CNTs‐S, CNTs/Gra‐S, and CNTs/Gra‐S‐Al_3_Ni_2_ cathodes at 1 C. b) Cycling performance of CNTs/Gra‐S‐Al_3_Ni_2_ electrode at current density of 2.76 mA cm^−2^ with ASLs of 3.30 mg cm^−2^. c) The digital photograph of three CNTs/Gra‐S‐Al_3_Ni_2_ batteries in series can light up 51 green indicators of 2835 LED modules (nominal voltage of 12 V and nominal power of 3 W).

The long‐term cycling performance of the three cathodes was evaluated at a rate of 1 C, with the results provided in **Figure** **5**a. Compared with the CNTs‐S and CNTs/Gra‐S cathodes, the unit incorporating Al_3_Ni_2_ exhibited a significantly higher cycle number. The CNTs/Gra‐S‐Al_3_Ni_2_ cathode delivered an excellent initial discharge capacity of 885 mA h g^−1^ and, after 800 cycles, the specific capacity was greater than 496 mA h g^−1^ with only a 0.055% average capacity decay per cycle. The CNTs/Gra‐S‐Al_3_Ni_2_ cathode also showed the highest coulombic efficiency, indicating that the notorious shuttle effect was effectively suppressed and that Al_3_Ni_2_ can improve cycling stability.

To satisfy the requirements of hybrid electric vehicle (HEV) and electric vehicle (EV) batteries, it has been suggested that the ASLs should be greater than 3 mg cm^−2^.^42^ As shown in Figure 5b, a CNTs/Gra‐S‐Al_3_Ni_2_ electrode with a 3.30 mg cm^−2^ sulfur mass loading exhibited a high initial areal capacity of 3.58 mA h cm^−2^ (1084 mA h g^−1^) at a current density of 1.11 mA cm^−2^. In addition, a high reversible areal capacity of 2.05 mA h cm^−2^ (622 mA h g^−1^) was maintained after 200 cycles, at an elevated current density of 2.76 mA cm^−2^, giving a superior capacity retention value of 85.9%. A comparison of the cycling performance observed in the present work with data for Li–S batteries from recent studies using sulfur loadings higher than 3 mg cm^−2^ is presented in Table S2 (Supporting Information).

To further explore the potential of CNTs/Gra‐S‐Al_3_Ni_2_ cathodes for practical high‐power applications, three half‐cells incorporating 3.25 mg of S were assembled in series. As shown in Figure 5c, with an open‐circuit‐potential of 7.05 V, this battery pack could efficiently drive 51 LED modules (at a nominal voltage of 12 V and nominal power of 3 W). These results clearly demonstrate the outstanding high‐power performance of the CNTs/Gra‐S‐Al_3_Ni_2_ cathode, as well as the feasibility of practical applications.

**Figure 6:**
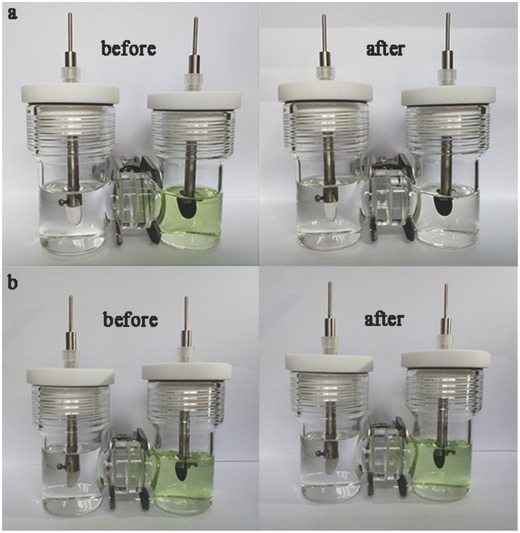
Digital photograph of the H‐type simulation electrolytic cell CV tests. a) CNTs/Gra/Al_3_Ni_2_ cathode before and after 150 CV cycles. b) CNTs/Gra cathode before and after 150 CV cycles.

The ability of Al_3_Ni_2_ to trap LiPSs was assessed in more detail using visible shuttle effect tests based on an H‐type simulation electrolytic cell with a central separator in conjunction with charging/discharging of the battery (**Figure** **6**). Both the left chambers were filled with pure electrolyte while the right chambers were filled with a LiPSs solution. We carried out CV trials with CNTs/Gra and CNTs/Gra/Al_3_Ni_2_ cathodes and a Li foil anode at room temperature within a potential window of 1.5–3.0 V at a sweep rate of 50 mV s^−1^ (Figure S8, Supporting Information). Initially, the color of the solution in the right side chambers was the same for all specimens. However, after 150 cycles, the color in the right chamber of the CNTs/Gra/Al_3_Ni_2_ unit was seen to fade (Figure 6a). In contrast, no distinct color change was observed in the case of the cell with the CNTs/Gra cathode (Figure 6b). These results provide direct evidence that Al_3_Ni_2_ effectively converted LiPSs and thus greatly suppressed the shuttle effect.

## 3 Conclusion

In summary, we successfully designed and fabricated a CNTs/Gra‐S‐Al_3_Ni_2_ cathode having a 3D network structure, and this cathode showed excellent cycling stability and high capacity retention. An experimental unit delivered a reversible capacity of 496 mA h g^−1^ over 800 cycles with an ultralow capacity degradation of 0.055% per cycle at 1 C. Simultaneously, a cathode made with a 3.30 mg cm^−2^ sulfur loading displayed a high reversible areal capacity of 2.05 mA h cm^−2^ (622 mA h g^−1^) at a higher current density of 2.76 mA cm^−2^ over 200 cycles in conjunction with a superior capacity retention of 85.9%. H‐type simulated electrolytic cell trials confirmed the ability of Al_3_Ni_2_ to promote LiPSs conversion reactions and thus suppress the shuttle effect during operation of a battery. The exceptional performance of this CNTs/Gra‐S‐Al_3_Ni_2_ cathode is attributed to its 3D network structure and the incorporation of Al_3_Ni_2_, which provides efficient channels for rapid electron and ion transfer, especially along the vertical direction of the cathode plane. These encouraging results obtained with Al_3_Ni_2_ suggest the possibility of developing high‐energy‐density and long‐cycle‐life Li–S batteries.

## 4 Experimental Section

*Synthesis of CNT/Gra*: The CNTs and Gra were purchased from a commercial corporation. The CNTs and Gra (the mass ratio = 2:1) were ultrasonically dispersed in the alcoholic solution for 120 min, and then the mixture was dried in the oven at 80 °C. The obtained material was called CNTs/Gra.

*Electrode Preparation*: GC electrodes (diameter of 3 mm, CH instrument Inc.) were polished in 0.05 and 0.3 µm alumina slurry (CH instrument Inc.) and then rinsed with ultrapure water and ethanol. Subsequently, the GC electrodes were ultrasonically cleaned in ultrapure water and ethanol, and finally dried under a gentle nitrogen stream. To prepare the working electrode, 2 mg CNT/Gra/Al_3_Ni_2_ (90 wt% Al_3_Ni_2_) was ultrasonically dispersed in 500 µL ethanol and deionized water (the volume ratio of ethanol and deionized water is 4:1), and 24 µL suspension was dropped onto the GC surface and dried at room temperature. For comparison, the CNTs/Gra electrode was prepared in the same way.

*Three‐Electrode Electrochemical Measurements*: The curves of CV were collected by an electrochemical station (CHI 760, Chenhua, China) at room temperature, where Pt wire, a Ag/AgCl electrode, and a CNTs/Gra/Al_3_Ni_2_‐modified GC electrode were used as the auxiliary electrode, the reference electrode, and working electrode, respectively.

*Synthesis of CNTs/Gra‐S Cathodes*: The CNTs/Gra‐S composites were prepared following a melt‐diffusion strategy. In a typical procedure, the CNTs and sulfur (high purity sulfur, 99.999% metal basis, Aladdin) were mixed according to the design of target composite. Then the powder was ground and heated in an oven at 160 °C for 12 h. From Figure S5a,b (Supporting Information), it is found that no bulk sulfur particles were formed, and the sulfur contents in this work were determined to be 65 wt%. The cathode for CNTs/Gra‐S Cathodes were prepared by mixing 85 wt% CNT/Gra‐S composite materials, 10 wt% conductive agent and 5 wt% polyvinylidene fluoride (PVDF) in 1‐methyl‐2‐pyrrolidinone (NMP) to form slurry. After stirring for 1 h, the slurry was pasted onto Al foil and dried at 55 °C overnight.

*Synthesis of CNTs/Gra‐S‐Al_3_Ni_2_Cathodes*: The cathode for Li–S batteries was prepared by mixing 75 wt% CNT/Gra‐S composite materials, 10 wt% conductive agent, 10 wt% Al_3_Ni_2_ powder and 5 wt% PVDF in NMP to form slurry. Subsequently, after stirring for 1 h, the slurry was pasted onto aluminum foil and dried at 55 °C overnight.

*Electrochemical Characterization*: Electrochemical experiments were performed via CR2025 coin‐type test cells assembled in an argon‐filled glove box with lithium metal as the counter and reference electrodes at room temperature. Celgard 2400 membrane was used as the separator to isolate electrons. The electrolyte was 1 m bis (trifluoromethane) sulfonimide lithium salt (LiTFSI) with 1% LiNO_3_ dissolved in a mixture of 1,3‐dioxolane and dimethoxyethane (1:1 by volume). The amount of electrolyte in the coin cell with the ASLs of 0.80 mg cm^−2^ is 50 µL. When the ASL is increased to 3.30 mg cm^−2^, the corresponding amount of electrolyte is 200 µL. The discharge/charge measurements were conducted using a Neware battery tests system (Neware Technology Co.). Before testing, the cells were aged for 24 h. CV and EIS measurements were performed on CHI660D electrochemical workstation. The scan rate for CV measurements was set as to be 0.1 mV s^−1^, and the DC voltage was kept at open‐circuit voltage and an AC voltage of 5 mV in amplitude was applied with a frequency of 200 kHz to 20 mHz in EIS measurements.

*Structure Characterization*: X‐ray diffraction patterns were obtained with a D/MAX‐2400 diffractometer using Cu Kα radiation (40 kV, 100 mA, λ = 1.54056 Å). SEM images were obtained with a JSM‐6700F field‐emission scan electron microscope. Thermo gravimetric analysis was measured with aSTA449 F3 Jupiter thermo gravimetric analyzer (NETZSCH), at a heating rate of 10 °C min^−1^ in nitrogen atmosphere. UV−vis absorption spectroscopy was use to characterize the polysulfide species and their contents in the electrolyte after the three‐electrode CV tests.

## Conflict of Interest

The authors declare no conflict of interest.

## Supporting information

Supplementary
