# Supplementary material for: 3D CNTs/Graphene‐S‐Al3Ni2 Cathodes for High‐Sulfur‐Loading and Long‐Life Lithium–Sulfur Batteries
Source: Adv Sci (Weinh). 2018 May 10;5(7):1800026. doi: 10.1002/advs.201800026 (PMC6051211; doi:10.1002/advs.201800026)
Supplement: Supplementary file 1 — Supplementary [file ADVS-5-1800026-s001.pdf]

Copyright WILEY-VCH Verlag GmbH & Co. KGaA, 69469 Weinheim, Germany, 2018.

ADVANCED SCIENCE
Open Access

Supporting Information

for *Adv. Sci.,* DOI: 10.1002/advs.201800026

3D CNTs/Graphene-S-Al_3Ni_2 Cathodes for High-Sulfur-Loading and Long-Life Lithium–Sulfur Batteries

Zeqing Guo, Huagui Nie, Zhi Yang,* Wuxing Hua, Chunping Ruan, Dan Chan, Mengzhan Ge, Xi'an Chen, and Shaoming Huang**

Submitted to ADVANCED SCIENCE

DOI: 10.1002/ ((please add manuscript number))

Three-Dimensional CNTs/Graphene-S-Al_3Ni_2 Cathodes for High-Sulfur-Loading and Long-Life Lithium-Sulfur Batteries

Zeqing Guo,Huagui Nie, Zhi Yang*, Wuxing Hua, Chunping Ruan, Dan Chan, Mengzhan Ge, Xi'an Chen, ShaomingHuang**

[*] Z. Guo, Prof. H. Nie, Prof. Z. Yang, W. Hua, C. Ruan, D. Chan, M. Ge, Dr. X. Chen, Prof.S. Huang

Nanomaterials & Chemistry Key Laboratory, Wenzhou University, Wenzhou, 325027,China

School of Material and Energy, Guangdong University of Technology, Guangzhou,510006, China

E-mail: huaguinie@126.com;yang201079@126.com;smhuang@wzu.edu.cn

Supporting Information

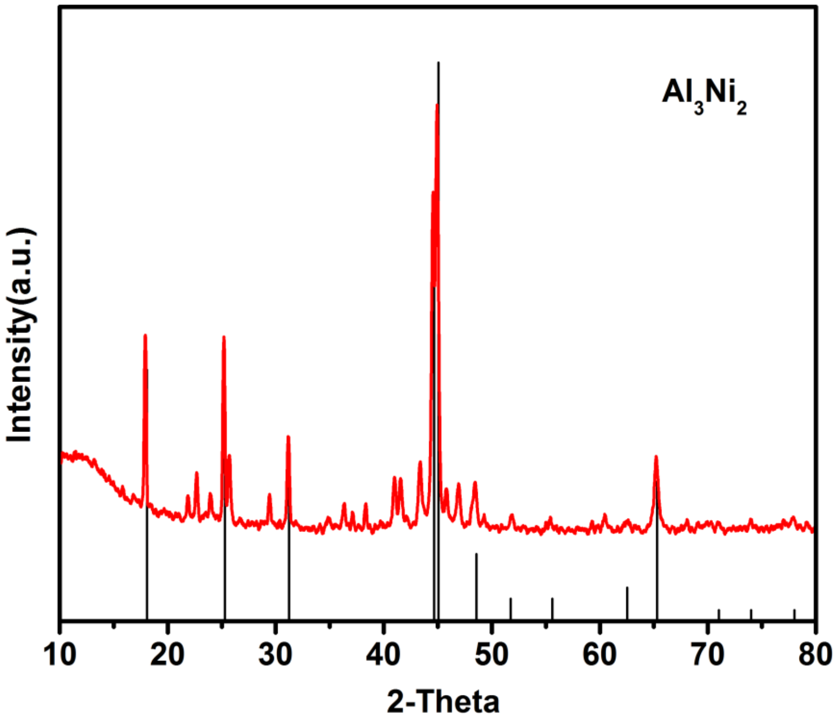

Figure S1. XRD patterns of Al_3Ni_2 .

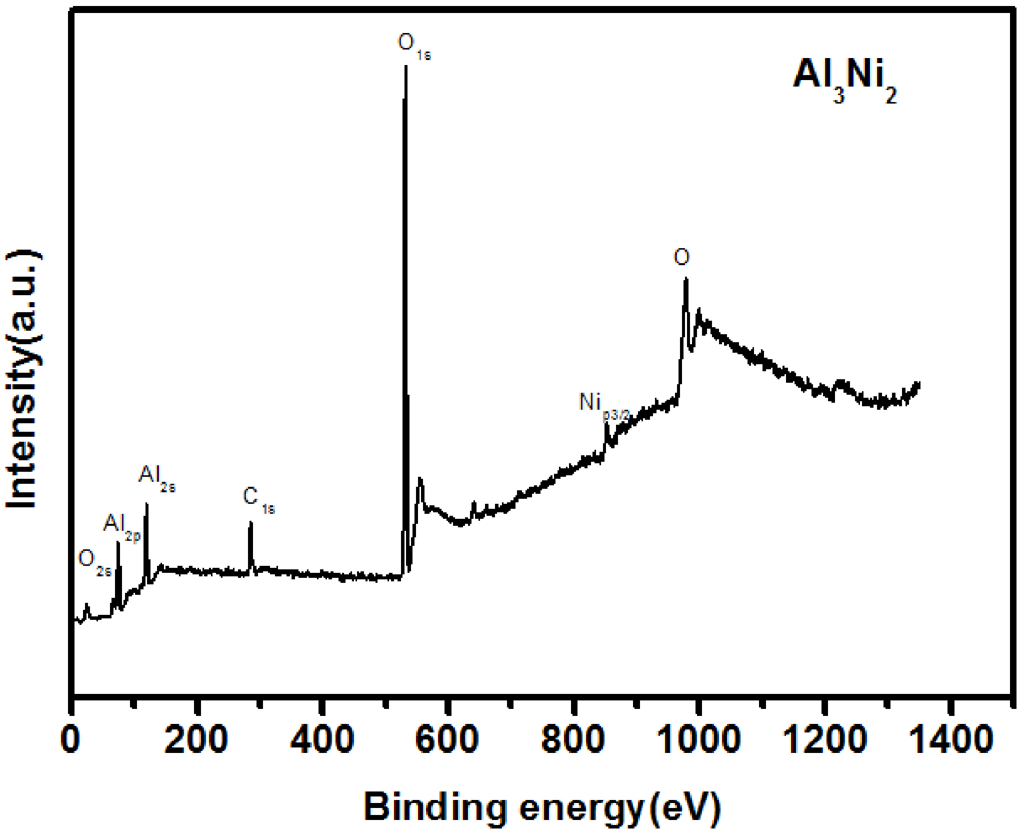

Figure S2. XPS spectra survey of theAl_3Ni_2.

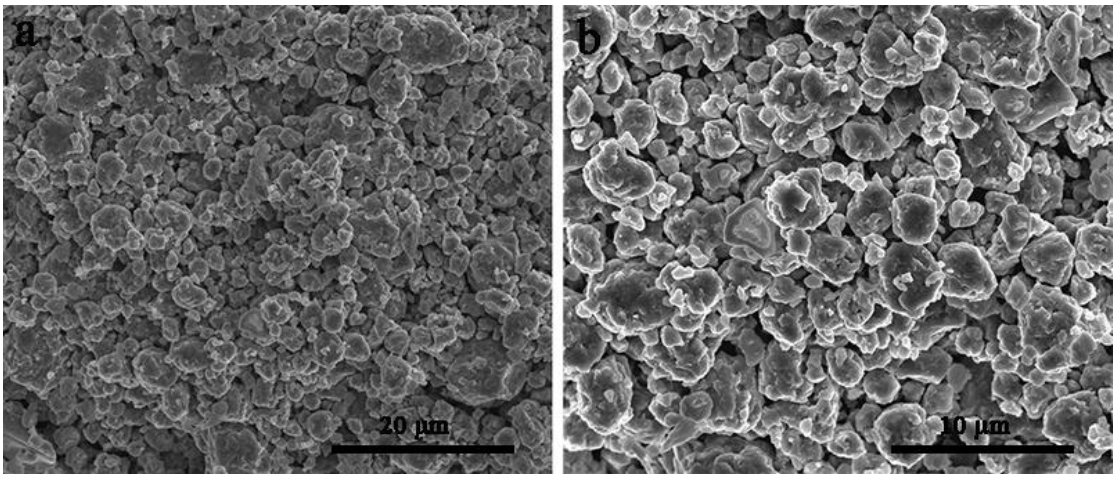

Figure S3. (a,b)SEM images of Al_3Ni_2.

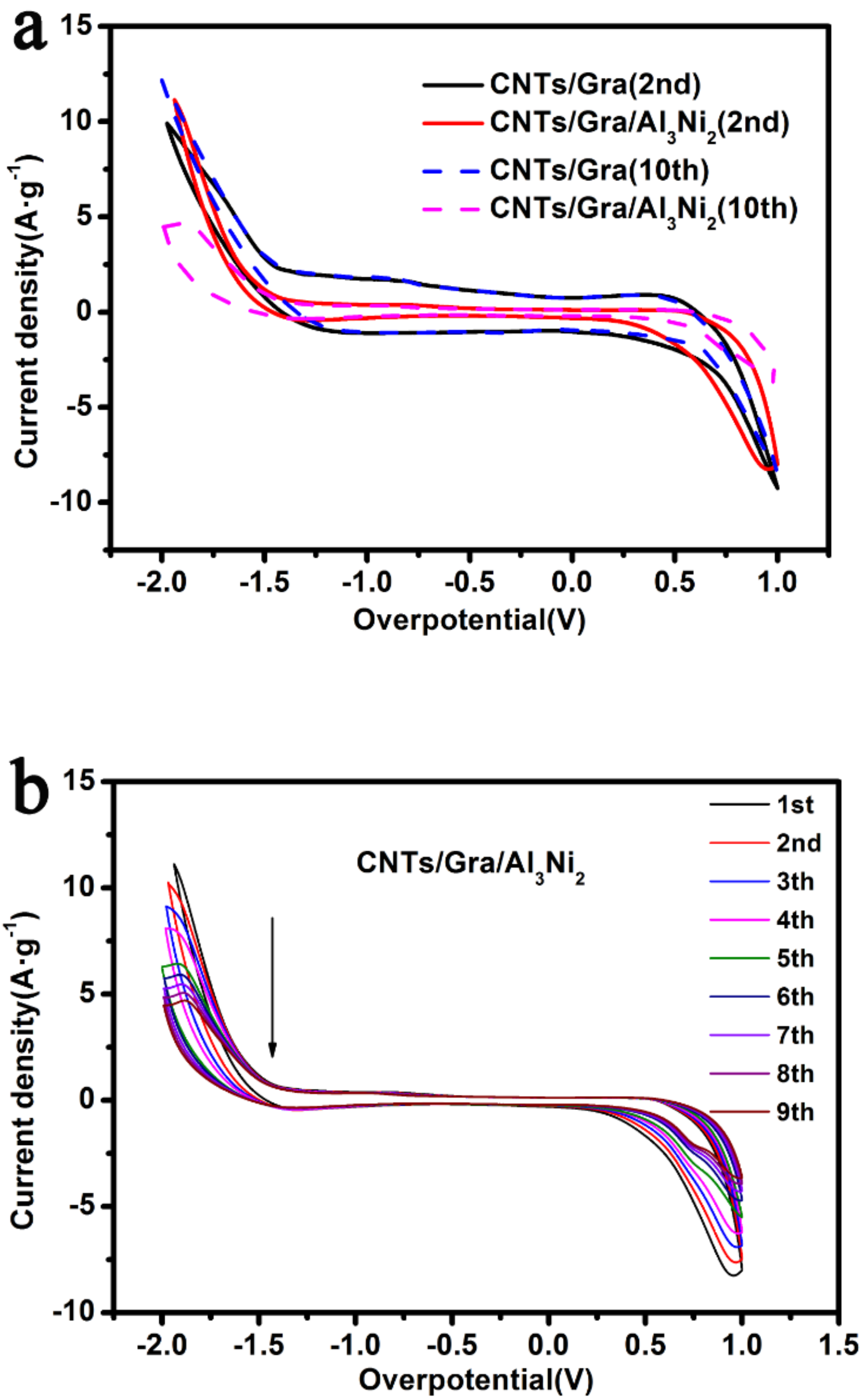
a
15
10
5
0
-5
-10
Current density(A·g-1)
-2.0
-1.5
-1.0
-0.5
0.0
0.5
1.0
Overpotential(V)
CNTs/Gra(2nd)
CNTs/Gra/Al3Ni2(2nd)
CNTs/Gra(10th)
CNTs/Gra/Al3Ni2(10th)
b
15
10
5
0
-5
-10
Current density(A·g-1)
-2.0
-1.5
-1.0
-0.5
0.0
0.5
1.0
1.5
Overpotential(V)
CNTs/Gra/Al3Ni2
1st
2nd
3th
4th
5th
6th
7th
8th
9th

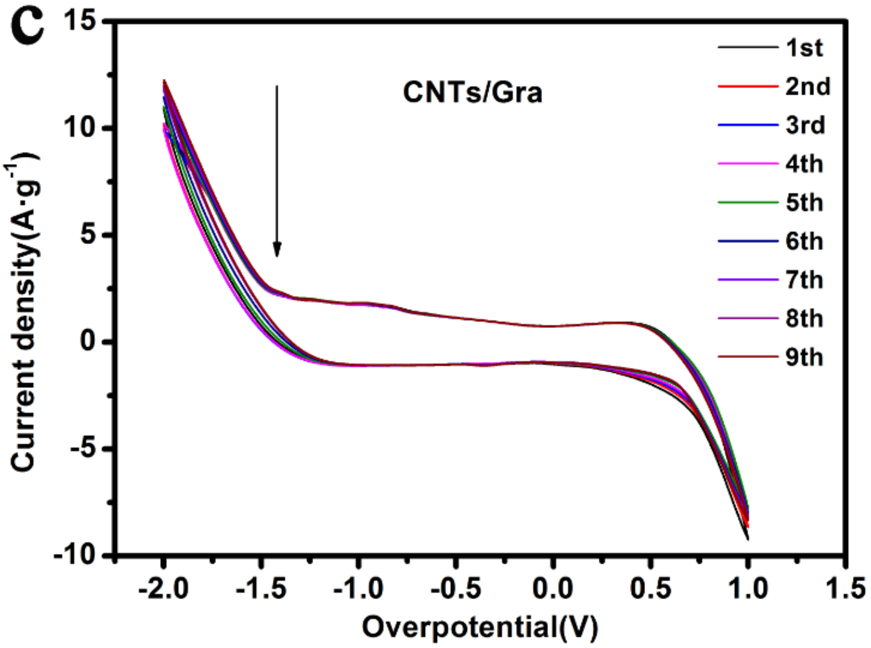

Figure S4. Three-electrode CV test. (a) The second and tenth cycle of the CV plots for the two electrodes. (b) CV plots of CNTs/Gra/Al_3Ni_2 electrode for 9 cycles. (c) CV plots of CNTs/Gra electrode for 9 cycles.

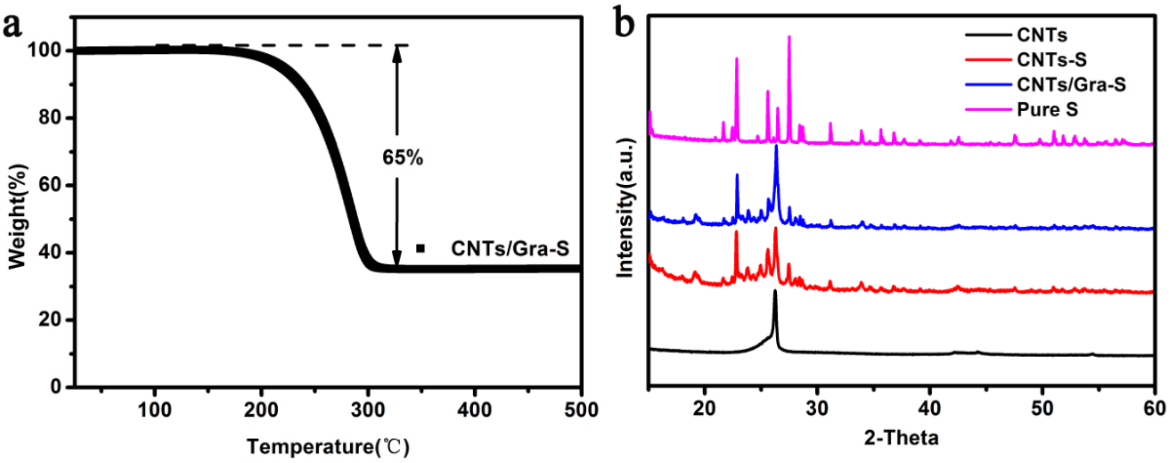

Figure S5. (a) TGA curve of the CNTs/Gra-S hybrids. (b) XRD patterns of puresulfur, CNTs, CNTs-S and CNTs/Gra-S hybrids.

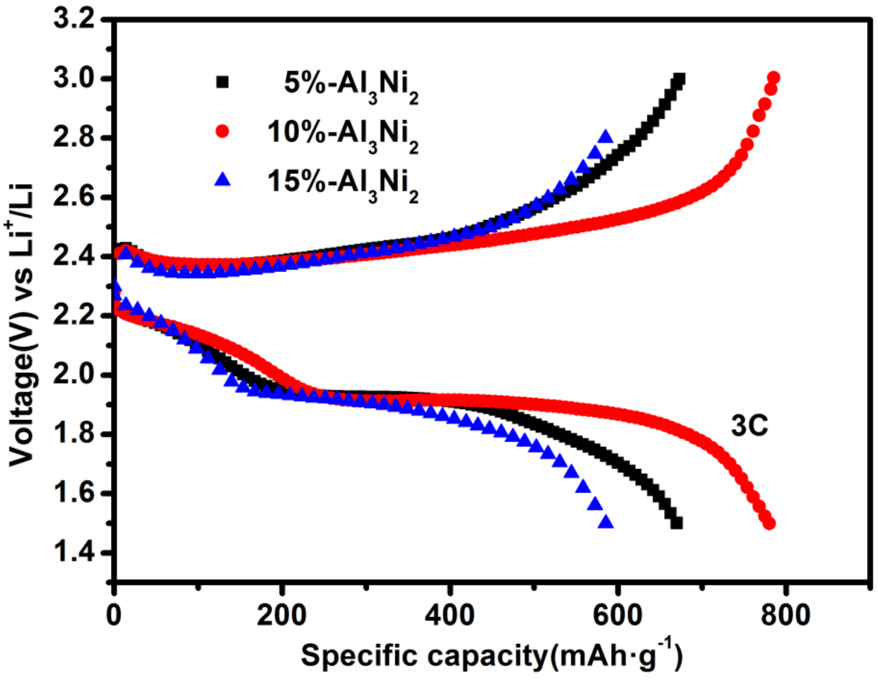

Figure S6. Typical galvanostatic charge-discharge profiles of CNTs/Gra-S-Al_3Ni_2 cathode for the second cycle at 3C with different content of Al_3Ni_2.

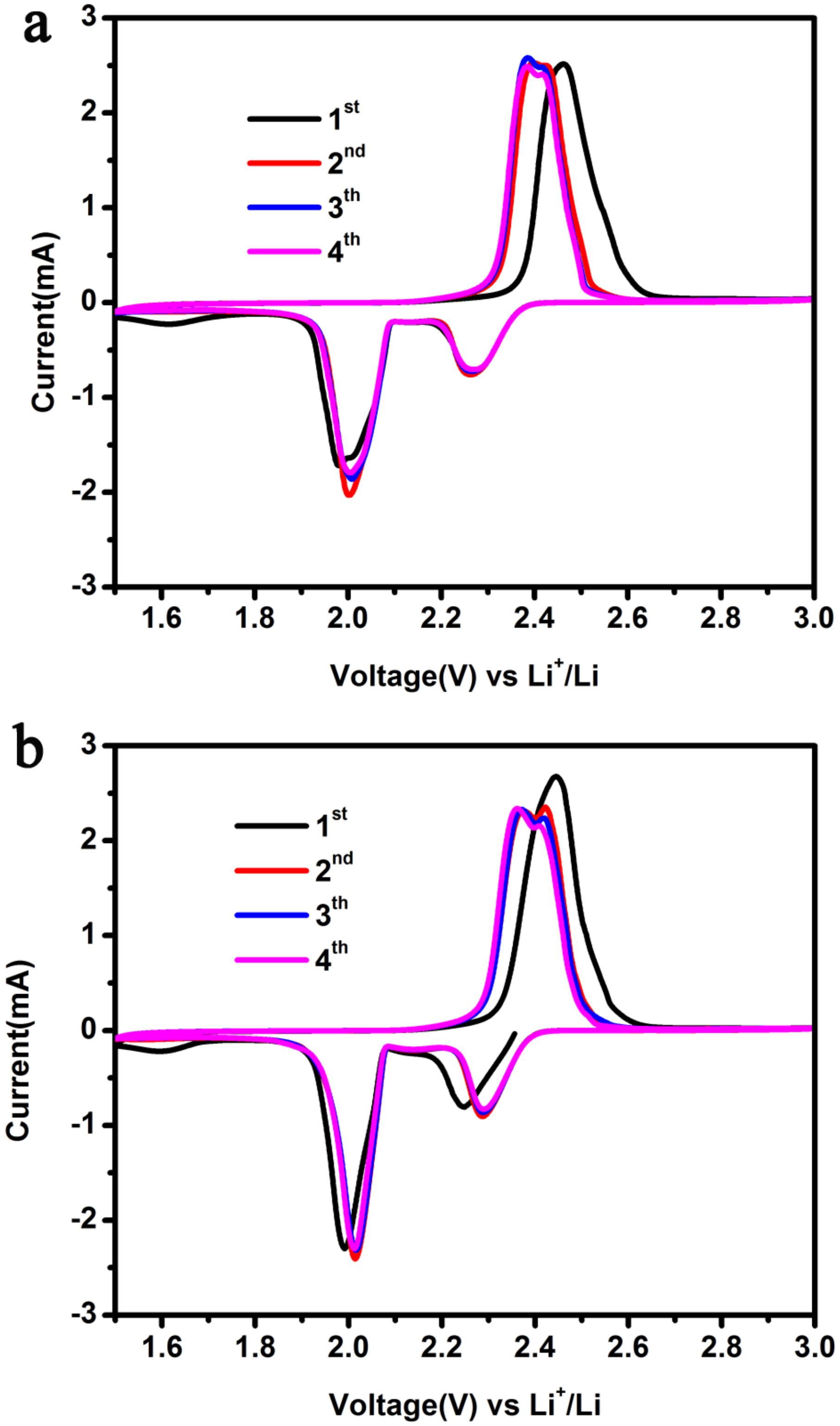

Figure S7. (a,b) The first four cycles of cyclic voltammetry curves for CNTs-S cathode and CNTs/Gra –S cathode respectively.

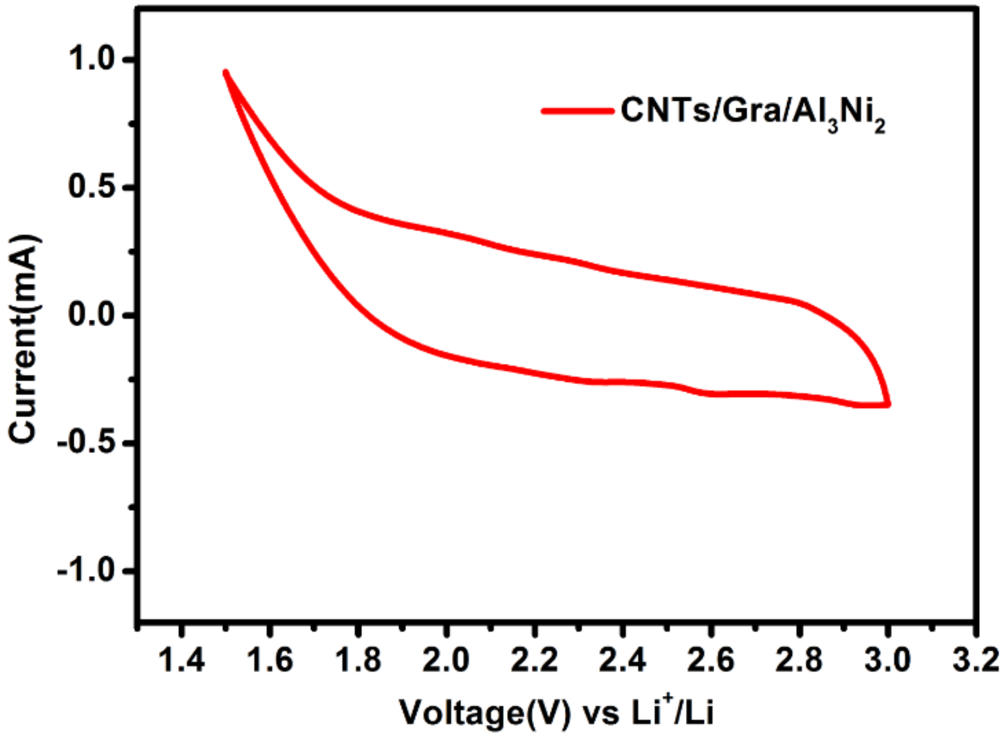

Figure S8. Polarization curves of H type glass cell with CNTs/Gra/Al_3Ni_2 cathode.

Table S1. Collection coeffient and voltage hysteresis of the three cathodes

Battery	collection coefficient	voltage hysteresis
CNTs/Gra-S-Al_3Ni_2	2.75	0.35
CNTs/Gra-S	2.66	0.37
CNTs-S	2.63	0.40

Table S2. Characteristics of various carbon-sulfur composites reported in literatures

Materials	S loading	Cycling performance	Capacity retention rate
TiC@G[1]	3.5mg cm^{-2}	0.2C,100cycles,1032~670mAhg^{-1}	64.9%
LDH@NG[2]	4.3 mg cm^{-2}	1.0mAcm^{-2},100cycles , 1078-800mAhg^{-1}	74.2%
LDH/S[3]	3.0 mg cm^{-2}	0.1C, 100 cycles, 1014~653mAhg^{-1}<br>0.5C, 100 cycles, 747~491mAh g^{-1}	64.4%<br>65.7%
CNTs-nest-85%S[4]	3.0 mg cm^{-2}	0.1C,80 cycles, 937-800mAh g^{-1}	85.3%
PCNTs-S@Gra/DTT[5]	3.51 mg cm^{-2}	1.17mA cm^{-2} (0.2C), 200 cycles, 1253~984mAh g^{-1}	78.5%
This work	3.30 mg cm^{-2}	2.76mA cm^{-2}(0.5C), 200cycles,724~622mAhg^{-1}	85.9%

References

[1]H. J. Peng, G. Zhang, X. Chen, Z. W. Zhang, W. T. Xu, J. Q. Huang, Q. Zhang.*Angew. Chem. Int. Ed.*,**2016**, 55, 12990.

[2] H. J. Peng, Z. W. Zhang, J. Q. Huang, G. Zhang, J. Xie, W. T. Xu, J. L. Shi, X. Chen, X. B. Cheng, Q. Zhang. *Adv. Mater.*, **2016**, 28, 9551.

[3]J. T. Zhang, H. Hu, Z. Li, X. W. Lou. *Angew. Chem. Int. Ed.*, 2016, 55, 3982.

[4]G. Ai, Y. L. Dai, W. F. Mao, H. Zhao, Y. B. Fu, X. Y. Song, Y.F. En,V. S. Battaglia, V. Srinivasan,G. Liu.*Nano Lett.*,**2016**, 16, 5365.

[5] W. X. Hua, Z. Yang, H. G. Nie, Z. Y. Li, J. Z. Yang, Z. Q. Guo, C. P. Ruan, X. A. Chen, S. M. Huang. *ACS Nano*, **2017**, 11, 2209.
